# Prediction and Sensitivity Analysis of Bubble Dissolution Time in 3D Selective Laser Sintering Using Ensemble Decision Trees

**DOI:** 10.3390/ma12091544

**Published:** 2019-05-10

**Authors:** Hai-Bang Ly, Eric Monteiro, Tien-Thinh Le, Vuong Minh Le, Morgan Dal, Gilles Regnier, Binh Thai Pham

**Affiliations:** 1 University of Transport Technology, Hanoi 100000, Vietnam; 2 PIMM, ENSAM, CNRS, CNAM, HESAM Université, 151 Boulevard de l’Hôpital, 75013 Paris, France; Eric.MONTEIRO@ensam.eu (E.M.); Morgan.DAL@ensam.eu (M.D.); gilles.regnier@ensam.eu (G.R.); 3 Institute of Research and Development, Duy Tan University, Da Nang 550000, Vietnam; 4 NTT Hi-Tech Institute, Nguyen Tat Thanh University, Ho Chi Minh City 700000, Vietnam; vuongminhle09@gmail.com

**Keywords:** 3D selective laser sintering, artificial intelligence, decision trees, bubble dissolution time, sensitivity analysis

## Abstract

The presence of defects like gas bubble in fabricated parts is inherent in the selective laser sintering process and the prediction of bubble shrinkage dynamics is crucial. In this paper, two artificial intelligence (AI) models based on Decision Trees algorithm were constructed in order to predict bubble dissolution time, namely the Ensemble Bagged Trees (EDT Bagged) and Ensemble Boosted Trees (EDT Boosted). A metadata including 68644 data were generated with the help of our previously developed numerical tool. The AI models used the initial bubble size, external domain size, diffusion coefficient, surface tension, viscosity, initial concentration, and chamber pressure as input parameters, whereas bubble dissolution time was considered as output variable. Evaluation of the models’ performance was achieved by criteria such as Mean Absolute Error (MAE), Root Mean Squared Error (RMSE) and coefficient of determination (R^2^). The results showed that EDT Bagged outperformed EDT Boosted. Sensitivity analysis was then conducted thanks to the Monte Carlo approach and it was found that three most important inputs for the problem were the diffusion coefficient, initial concentration, and bubble initial size. This study might help in quick prediction of bubble dissolution time to improve the production quality from industry.

## 1. Introduction

Selective laser sintering (SLS) is one of the most important 3D printing technique widely used in industry [1]. The main idea of this method is that powders are sintered by laser in order to produce solid objects predefined by 3D sketch models [2]. The addictive manufacturing technique exhibits many interesting behaviors not found in traditional methods. For instance, using 3D sketches combined with laser sources, SLS technology could easily handle complex geometries of objects [3,4,5,6,7]. Secondly, laser can melt a lot of materials such as metal, ceramic, polymer or glass powders, especially in case of polymeric particles [8,9,10,11]. In industry of polymer processing, the SLS method exposure also has great advantages, particularly in terms of saving time in fabrication [12]. However, combining particles and laser technique requires broadly information involving many multiphysical phenomena. For instance, depend on the nature of polymeric material, relationship between many parameters such as particle granulometry and morphology, laser power, and scan speed should be established in order to improve the production quality. Unfortunately, solid structures created using SLS technique are under the presence of many imperfections such as porosities, cracks, or lacks of fusion [12,13]. The existence of these defects could be explained by the complexity and the coupling of the different physics occurring in the process, for instance melting, coalescence, gas diffusion, and crystallization [14,15,16].

One of the most important defects using SLS technology is the formation of gas bubbles in fabricated parts [8]. As an illustration, for a considered layer during the process, laser beam irradiates polymer particles spread over the previous layer. Gas bubbles are therefore trapped in the melted layer by the difference between resulting temperatures [1]. The larger the initial size of the bubbles, the more dissolution time requires. That means the understanding of dissolution time of gas bubbles has a crucial impact on the processing of solids using SLS technique. However, it is a complex problem which depends on many factors, such as bubble internal radius, external radius, diffusion coefficient, surface tension, viscosity, saturation, and pressure [17]. Until now, no known analytical work has been proposed to predict such bubble dissolution time. Only semi-analytical or numerical methods have been established for investigating the influence of parameters on dissolution time. For instance Kontopoulou et al. [8] studied the impact of viscosity, initial bubble size, and applied pressure in order to determine the kinetic of bubble shrinkage. In another work, surface tension, diffusion coefficient, and saturation of polymer melt have been integrated to establish an equation of bubble dissolution time [18]. Many other attempts have also been introduced in order to relate the dissolution of bubbles and physical constant of the problem [19,20,21].

In general, there are three techniques commonly used in the literature to predict the mechanical properties of materials: computational modeling and simulation, experimental studies, or artificial intelligence (AI) approach. The modeling and simulation of physical–mechanical phenomena is usually complicated, requires extensive computation time, and is thus not widely used in practice. Experimental studies are costly, time consuming, and required a lot of specific equipment. Out of these, the artificial intelligence techniques have been recently used in many fields of science [22,23,24]. This technique exhibits a lot of advantages not found in traditional computational simulation [25], especially for predicting material behaviors, for instance, rheological properties of materials [26], permeability of polymers [27], composite materials [28], structure under compression [29], or concrete [30]. Nevertheless, to the best of our knowledge, no artificial intelligence approaches have been applied to predict bubble dissolution time in the SLS process as well as quantify the relationship between physical parameters of the problem. Moreover, works related to the modeling of bubble dissolution process only consider an infinite melted medium [8,18], which is not the case in SLS technique due to the entrapped gas bubble are close to the surface of powder bed. A proper approach is needed to take into account the finite medium in SLS process. In addition, laboratory experiments could only be used to observe the growth and collapse phenomena [31] but not the factors that affect the mechanism. The approach using AI algorithms, once developed, could provide efficient information and help to improve the quality of SLS production.

This contribution applied two AI algorithms namely the Ensemble Bagged Trees (EDT Bagged) and Ensemble Boosted Trees (EDT Boosted) to predict bubble dissolution time. The outline of the present study is organized as follows. The Introduction (Section 1) starts with the state-of-the-art of the problem. Section 2 (Materials and Methods) is devoted to describing the mechanism of the bubble shrinkage problem and the construction of data used for AI modeling. Bubble initial radius, external domain radius, diffusion coefficient, surface tension, viscosity, saturation, and chamber pressure were considered as inputs; and bubble dissolution time is output for the prediction tools. A brief introduction of two AI techniques based on the Ensemble Decision Trees algorithms is presented next, together with the Monte Carlo approach for the input sensitivity analysis. Principal results and discussions are presented in Section 3 (Results and Discussions), including the prediction capability of each model along with the sensitivity analysis of input parameters. Finally, the paper is completed with several conclusions and perspective in Section 4 (Conclusions).

## 2. Materials and Methods

### 2.1. Physical Problem and Data Preparation

#### 2.1.1. Mechanical Description of the Bubble Dissolution Process

When fabricating parts in the SLS process, grains coalesce faster at the surface than deeper in the powder bed because of higher laser radiation absorption at its surface. Air porosities are then embedded in the melted layers, whose thickness can reach a few hundred of microns. Air porosity, or air bubbles, is an essential factor accounting for the final mechanical properties of the fabricated parts. Thus, understanding bubble dissolution process in the melted polymer is important, especially in SLS technique.

Bubble dissolution mechanism is a highly coupled and nonlinear physical problem. Noting that proper development of equations has been presented in our previous work, in this paper, only principal equations that have impact on the AI modeling part will be briefly recalled. The nature of the resorption process lied on the diffusion of gas inside the bubbles (the gas phase) into the melted materials, or the liquid phase, such as metal, glass, or polymer. During the resorption process, the movement of the interface between gas/liquid phases obeyed the fluid dynamics equations, represented by the equation of conservation of momentum [17]:

(1) $$[P_{b}(t)-P_{c}(t)]R_{i}(t)-2\gamma \left(1+\frac{R_{i}(t)}{R_{e}(t)}\right)+4\eta \left(\frac{R_{i}^{3}(t)}{R_{e}^{3}(t)}-1\right){\dot{R}}_{i}(t)=0$$

where *P_b_*(*t*) and *P_c_*(*t*) represent the pressure of the perfect gas inside the bubble and the chamber of the SLS machine, respectively; *R_i_*(*t*), *R_e_*(*t*) denotes the bubble initial radius and the radius of the outer liquid phase; ${\dot{R}}_{i}(t)$ is the rate of bubble dissolution; *η* refers to the fluid viscosity and *γ* stands for the surface tension. The liquid phase is characterized by fluid viscosity, whereas the surface tension depends on both media as it is resulted at the air-liquid interface. Besides, the rate of gas diffusion is related to the movement of the interface through the diffusion equation, or the Fick law [17]:

(2) $$\frac{\partial c}{\partial t}=\frac{D}{r^{2}}\frac{\partial }{\partial r}(r^{2}\frac{\partial c}{\partial r})$$

where *D* stands for the diffusion coefficient, *c* is the concentration of gas in the fluid phase. The degree of saturation is introduced in order to take into account the boundary conditions derived from Henry’s law such as [17]:

(3) $$c(r,0)=x_{sat}K_{H}P_{C}(0)$$

with *K_H_* the Henry’s law constant. Finally, the mass balance equation at the bubble interface, in considering the decrease of perfect gas within the bubble is proportional to the flux across the interface, reads [17]:

(4) $$\frac{\partial }{\partial t}\left(\frac{P_{b}(t)R_{i}^{3}(t)}{R_{g}T}\right)=3R_{i}^{2}(t)D{\frac{\partial c(r,t)}{\partial r}|}_{r=R_{i}}$$

with *R_g_* the universal gas constant and *T* the temperature. For summary, the dissolution mechanism depends on the nature of the fluids (the molten materials), the mechanism of gas diffusion (air) and the mass balance at the gas/fluid interface. In the aim of predicting bubble dissolution time in the selective laser sintering (SLS) process, the need of experimental values of physical constants (inputs) as well as results (targets) are important. However, to the best of our knowledge, very few existing investigations clearly determining such parameters have been reported so far. These values are extremely difficult to determine. Therefore, simulation data constructed from previously developed numerical tool [17] have been used in this investigation. In this work, a finite element-based numerical tool has been developed in order to model and explain the influence of diverse parameters on the dissolution time, such as initial bubble size (*R_i_*), the external radius of the fluid phase (*R_e_*), the surface tension (*γ*), viscosity (*η*), diffusion coefficient (*D*), initial melt concentration (*x_sat_*), and the chamber pressure (*P*). The fluid domain was assumed Newtonian. It is noteworthy that results extracted from such a numerical tool have already been compared and validated with experimental studies on the bubble removal in rotational molding process [8,18], a technique similar to the SLS.

#### 2.1.2. Factors that Affecting Bubble Dissolution Time

In this paper, the prediction capability and sensitivity analysis of physical parameters that may have influence on the dissolution process were performed. It is important noticed that a change of any parameter affects all others, which is governed by the dynamic, thermodynamics, or physics of the bubble shrinkage problem [17]. For example, the change of gas inside bubble or the polymer melt causes changes in viscosity, surface tension, and diffusion coefficient applied to both liquid and gas phases. Therefore, all the parameters were varied in a broad range in order to fully collect results and analyze their effect to the bubble dissolution time.

Input parameters considered for the AI simulation were bubble radius (I_1_), bubble external radius (I_2_), diffusion coefficient (I_3_), surface tension (I_4_), viscosity of the melted materials (I_5_), initial saturation (I_6_) and chamber pressure (I_7_). The prediction output was bubble dissolution time (O). The values of physical parameters, i.e., I_1_, I_2_, I_5_, (used in Equation (1)) were taken based on experimental data in the work of Kontopoulou et al. [8]. The diffusion coefficient (I_3_) was varied around that in the case of air diffuse into polyethylene melt (used in Equations (2) and (4)), which was estimated previously in the work of Griskey [9]. Surface tension values (I_4_) had been taken based on the case of polyethylene melts (used in Equation (1)), which was gathered from earlier study of Wu [32]. The viscosity of the melted polymer (I_5_—used in Equation (1)) was collected following the works of Bird et al. [33] and Durrill et al. [34]. Being used in Equation (3), the initial saturation (I_6_) and the chamber pressure (I_7_) were varied from that of the analytical study of Gogos [18]. Parameters such as the Henry’s law constant, air parameters or the temperature dependence needed for applying an Arrhenius equation were not considered as inputs, as they are often directly related to one or several inputs I_1_ to I_7_. Simulated data were taken to cover all the possibilities in the SLS process. A total number of 68,644 data were generated, lied on the fact that the corresponding dissolution time of these data varied from 20 s to 1200 s, which is of interest in the SLS process. If it is lower than 20 s, the dissolution process is quite quick and improvement of the SLS process appears less important. If it is higher than 1200 s, the dissolution process is too long so that it appears not interesting to be studied. A summary of the input and outputs values are presented in Table 1.

As shown in Table 1, the dissolution time of bubble considered in this study varied from 20 to 1200 s, with a mean value of 194.2 s and a standard deviation of 234.9 s. That means the distribution of bubble dissolution time was highly asymmetric and exhibited a big amount of quantity distributed between 20 s and 200 s. This interval of dissolution time was very important in the SLS processing industry. The lower dissolution time of bubbles, the higher production quality.

#### 2.1.3. Datasets

In general, the training and testing datasets are required for constructing and validation of AI models. Data were divided into 2 groups at a ratio of 70/30. A total number of 48,051 training data (70%) were used to construct the prediction models, whereas 20,593 testing data (30%) were used for evaluation and validation of the models. The 70/30 ratio for generating the datasets in this study has been done as suggested by Khorsheed and Mohammad [35], and Leema et al. [36]. Description of two AI algorithms in this study will be briefly presented in the next section.

### 2.2. Background of Models Used

#### 2.2.1. Decision Trees Methods

Decision trees (DT), proposed by Quinlan [37], is a popular machine learning method that can be applied in solving many real world problems such as identifying risk factors for drug use [38], flash flood prediction [39], landslide spatial prediction [40], prediction of a short-term photovoltaic power [41]. Main principle of the DT is to use a series of rules to identify the regions with the most homogeneous output variables to input variables on which a constant is fitted to each region. Main advantages of the DT are (i) it represents information in an intuitive and easy way for visualization, (ii) it is powerful for mining nonlinear and interactions effects between dependent and independent variables, (iii) it requires no mathematical assumptions between output and input variables, and (iv) it is capable to handle missing values and outliers [42]. On the other hands, the DT also faces several problems such as (i) it is difficult to model smooth functions, (ii) the structure of tree is sensitive with the sample data as only small change in training data can give very different results, and (iii) it has high variance and low bias [43]. Therefore, many techniques have proposed to enhance the predictive capability of the DT such as Ensemble Bagged Trees and Ensemble Boosted Trees.

##### Ensemble Bagged Trees (EDT Bagged)

EDT Bagged is a hybrid model which is a combination of bagging algorithm and decision trees [44]. Out of these methods, bagging method uses the bootstrap sampling to optimize the input training data for learning the decision trees [45]. This process can be carried out in several steps such as (i) it takes a bootstrap sample from the original training dataset, (ii) it fits the decision trees with the extracted dataset, (iii) it then finds the best fit models with the optimized dataset, (iv) it thereafter uses each of the fitted models with the optimize dataset to predict the results, and (v) it finally averages the prediction results of all the models [46]. EDT Bagged can significantly improve the prediction accuracy as bootstrap aggregation used in bagging method can reduced the variance in individual prediction methods like decision trees. EDT Bagged has been applied to solve many real world problems namely travel time prediction [44], disease prediction [45], and bankruptcy prediction [47]. In this study, we have used the EDT Bagged for prediction of bubble dissolution time when manufacturing solid structures by SLS technique.

Suppose that $x=\left(x_{1},x_{2},\ldots ,x_{n}\right)$ is a vector of input variables (*n* is the number of input variables), the bootstrap aggregation used in EDT Bagged to average the predictions is defined as follows [46]:

(5) $$f_{bag}\left(x\right)=\frac{1}{N}\sum_{i=1}^{N}f_{i}^{*}\left(x\right)$$

where *N* is the number of various training datasets generated by bootstrapping, and $f_{i}^{*}\left(x\right)$ are defined as predictors.

##### Ensemble Boosted Trees (EDT Boosted)

EDT Boosted is an additive regression model in which individual terms are simple trees, fitted in a forward, stagewise estimation [48]. It is well-known as an ensemble method which combines regression trees and boosting techniques in which the regression trees are used to connect output variables to input variables by recursive binary splits whereas the boosting technique is used to combine many single models to improve the predictive capability of the ensemble models [49]. EDT Boosted takes advantages of tree-based methods, and even overcomes the drawback of single tree models as (i) it is able to select relevant variables to fit accurate functions, (ii) it can be fitted with different amounts of data using stochastic boosting, and (ii) it can reduce both bias by forward stagewise fitting and variance by model averaging [48]. EDT Boosted has been applied to many fields such as banking [49], ecology [50], and medical [51]. In this study, EDT Boosted is a first time use for predicting the dissolution time of bubble removal when using laser to melt polymer powders in a SLS procedure.

Suppose that $x=\left(x_{1},x_{2},\ldots ,x_{n}\right)$ is a vector of predictors (*n* is the number of predictors) and y is a response, the EDT Boosted algorithm is trained by following function [52]:

(6) $$f\left(x\right)=\sum_{k}f_{k}\left(x\right)=\sum_{k}\alpha_{k}t\left(x,y_{k}\right)$$

where $\alpha_{k}$ values represent weights given to the nodes of each tree, $y_{k}$ is defined as the split variables, $t\left(x,y_{k}\right)$ represents single decision trees.

#### 2.2.2. Monte Carlo Method

**Figure 1:**
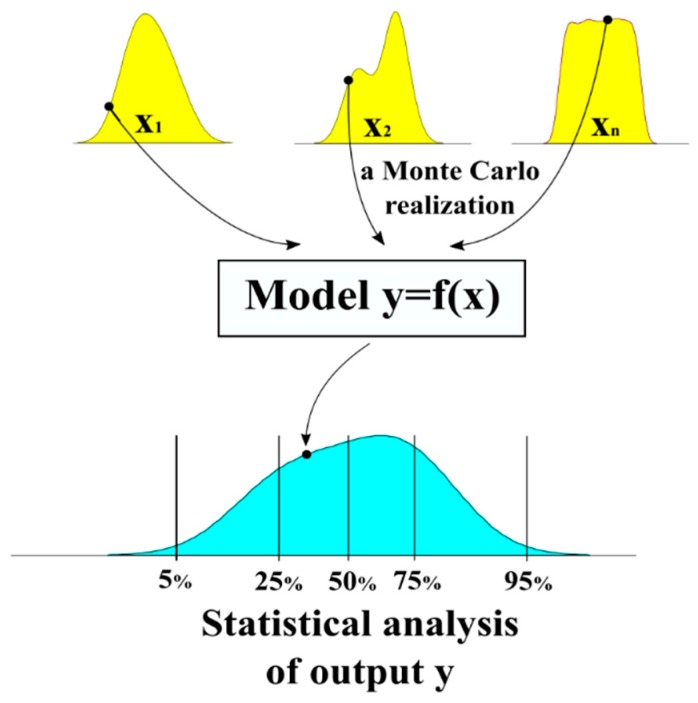
Schematization of using Monte Carlo method for the propagation of input variability.

In many fields of science, average values of parameters are not sufficient enough for investigating the problem and also the complex relationship between parameters. For instance, material variability and structural variability could largely impact the effective mechanical behaviors of composite structure [53,54,55,56]. Along with the development of computational algorithms and computer performance, Monte Carlo method appeared then as a highly efficient technique for accounting variability of parameters when they become relevant [57]. This method could help researchers to determine which parameters are important on the overall responses following variability of each input. Indeed, Monte Carlo method exhibits high capability to quantify numerically linear or non-linear relationship in data. The main idea of this method is to repeat random sampling of inputs using probability density distribution of each. By doing so, variability and of inputs could be fully propagated to the overall properties though constructed models. Statistical analysis of outputs is next carried out in order to quantify the impact of input variability on the responses. A schematization of using Monte Carlo method for propagating input variability is presented in Figure 1.

Monte Carlo method could also be used for analyzing the sensitivity of input parameters on the responses though constructed models [58]. For instance, the considered input has no influence on the final responses if it is removed from the input space and the final responses has statistically no differences compared to the case of simulation with all inputs. This way, the sensitivity of all inputs to the overall properties could be entirely quantified. This analysis has an important role for designer, for instance, dimension of the problem could be reduced and time consuming could be saved by eliminating insignificant parameters. To the contrary, sensitive parameters exposure important role and receive so huge attention.

In this study, the “normdiff” function of random variable P was introduced in order to: (i) determine the corresponding optimal number of Monte Carlo realizations and (ii) evaluate the degree of fluctuation of P around its average value (in % unit). The normdiff function was defined as [55]:

(7) $$\mathrm{normdiff}\left(N_{r}\right)=\frac{1}{\bar{P}N_{r}}\sum_{i=1}^{N_{r}}P_{i}$$

where N_r_ is the actual number of Monte Carlo runs and $\bar{P}$ is the average value of P. In this work, the normdiff was applied for estimating the statistical convergence of several random variables such as Root Mean Square Error (RMSE), Mean Absolute Error (MAE), and coefficient of determination (R^2^) respectively (see Section 2.2.3.).

Uniform distribution was applied to perform random sampling of input data for Monte Carlo simulations. Random subsets of input data were drawn uniformly between 1 and last sample index. The rectangular distribution ensures in this case no constraints for generating input realizations (i.e., all sub-spaces of the input space were accounted in the simulation).

#### 2.2.3. Validation Criteria

In this study, popular validation criteria namely Root Mean Square Error (RMSE), Mean Absolute Error (MAE), and coefficient of determination (R^2^) were selected for validating the predictive capability of ensemble decision trees [59,60]. RMSE measures the average squared difference between actual and predicted values, MAE measures the average magnitude of the errors, and R^2^ measures the correlation between actual and predicted values [59,60]. Whereas R^2^ varies from 0 (lowest models) and 1 (best models), higher RMSE and MAE shows lower predictive capability of the models. We can calculate these RMSE and MAE and R^2^ by following equations [61,62]:

(8) $$RMSE=\sqrt{\frac{1}{m}\sum_{i=1}^{m}{\left(v_{i}-{\bar{v}}_{i}\right)}^{2}}$$

(9) $$MAE=\frac{1}{m}\sum_{i=1}^{m}\left(v_{i}-{\bar{v}}_{i}\right)$$

(10) $$R^{2}=1-\frac{\sum_{i=1}^{m}{\left(v_{i}-{\bar{v}}_{i}\right)}^{2}}{\sum_{i=1}^{m}{\left(v_{i}-{\bar{v}}_{}\right)}^{2}}$$

where *m* is considered as the number of the samples, $v_{i},{\bar{v}}_{i},and\;\bar{v}$ are the actual output, the predicted output, and the mean of the ${\bar{v}}_{i}$, respectively.

#### 2.2.4. Methodology Chart

**Figure 2:**
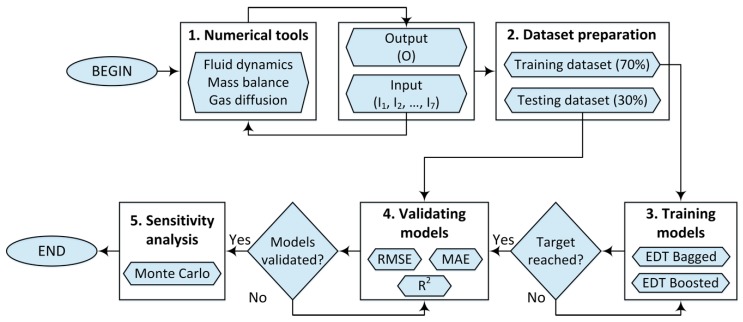
Methodology of the proposed analysis.

We present here different steps of the present work’s methodology, which contains five main steps as followings (as shown in Figure 2):

**Step 1**. Numerical tools for collecting data. In this step, all input parameters such as bubble initial radius (I_1_), bubble external radius or the domain size (I_2_), the diffusion coefficient (I_3_), surface tension (I_4_), viscosity of the melted materials (I_5_), the initial saturation (I_6_), and the chamber pressure (I_7_) were used to compute the prediction output-bubble dissolution time (O), using the developed finite element model [17]. Fluid dynamics, mass balance and gas diffusion were taken into account in this numerical model.

**Step 2**. Dataset preparation for training AI models. In this step, all input and output parameters were used to create a complete set of data. A number of 70% data (48,051 training data) were taken from the initial dataset for training the two proposed AI models. The remaining 30% data (20,593 testing data) were used for validating step, after the AI models were trained.

**Step 3**. Training models. In this step, the AI models were trained using the training data set. Two AI algorithms were used: EDT Bagged and EDT Boosted. The algorithm of these two models were introduced in the previous section. This step was repeated until the models were successfully trained (a tolerance error criteria was reached).

**Step 4**. Validating models. In this step, after the two AI models were trained, the validation process was performed using the testing data set (30% of the initial data set). The models were validated using different statistical measures such as: Root Mean Squared Error (RMSE), Mean Absolute Error (MAE) and the coefficient of determination (R^2^).

**Step 5**. Sensitivity analysis. After validation of the two proposed AI models using the testing data set, the sensitivity of input parameters was performed. This process was carried out thanks to the Monte Carlo approach.

## 3. Results and Discussion

### 3.1. Comparison of Ensemble Decision Trees (EDT) Algorithms

**Figure 3:**
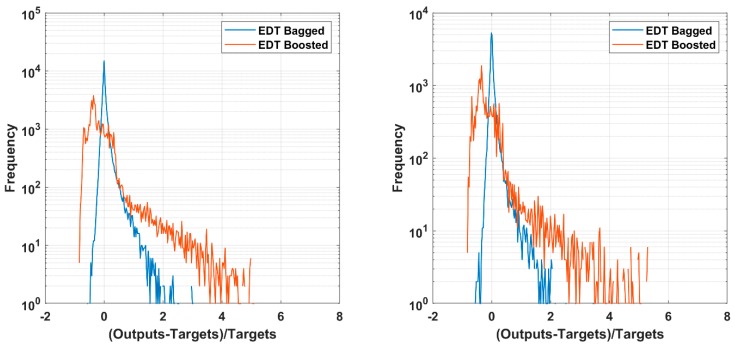
Histogram of relative error between predicted and output values of training (**left**) and testing (**right**) part of Ensemble Bagged Trees (EDT Bagged) and Ensemble Boosted Trees (EDT Boosted) algorithms.

Validation of the two proposed AI models are illustrated in Figure 3 for the training part including 48,051 data (left) and testing part with 20,593 data (right). Error frequency are plotted in function of relative errors between target and output values for EDT Bagged (blue line) and EDT Boosted (orange line) algorithms. In both training and testing parts, the EDT Boosted exhibit more error between output and target, as the values ranging from −1 to 6. The predicted outputs are ranging from 1 time lower to 6 times higher than the target ones. As regard to the training part, the over predicted values outside the range −1 to 1 were 1880 samples (or 3.9%) for EDT Boosted and 275 samples (or 0.57%) for EDT Bagged. For the testing part, the over predicted values outside the range −1 to 1 were 893 samples (or 4.3%) for EDT Boosted and 163 samples (or 0.79%) for EDT Bagged, showing reasonable performance of the AI models. Considering the EDT Bagged algorithm, the error range between output and target is rather narrower than the EDT Boosted model, i.e., ranging from −0.5 to 2. The peaks (blue line) of both training and testing parts are centered at 0, showing excellence prediction capability of EDT Bagged. For both AI algorithms, the number of over predicted values are more than that of under predicted values, confirmed by the error curves are rather on the right side of 0. It is worth noticed that for this simulation, the error measurements are: RMSE = 24.38, MAE = 9.70, R^2^ = 0.989 for training EDT Bagged, RMSE = 28.28, MAE = 9.91, R^2^ = 0.986 for testing EDT Bagged, whereas these values are RMSE = 141.48, MAE = 79.64, R^2^ = 0.852 for training EDT Boosted, RMSE = 144.80, MAE = 81.80, R^2^ = 0.843 for testing EDT Boosted. In general, both of methods show good predictive capability based on the validation criteria R^2^, RMSE and MAE. Comparing the prediction performance using testing parts, EDT Bagged appears a better candidate for this problem, as the RMSE, MAE, and the number of wrong predictions are smaller than those of EDT Boosted, whereas the R^2^ values are higher than that of the EDT Boosted model.

**Figure 4:**
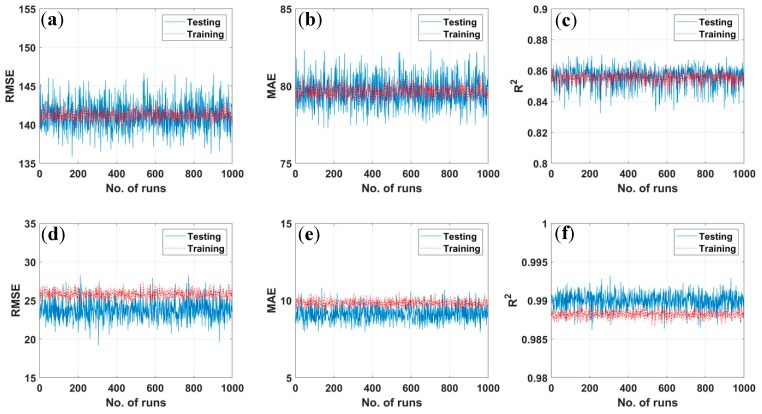
Error distribution (Root Mean Squared Error (RMSE), Mean Absolute Error (MAE), and coefficient of determination (R^2^)) of different models for 1000 simulations: (**a**–**c**) EDT Boosted and (**d**–**f**) EDT Bagged algorithms.

In validation stage, it is difficult to conclude the performance of given AI algorithms unless a fully analysis is performed. As 70% of data are randomly taken to train the AI prediction tools, the corresponding performance measurements (RMSE, MAE, or R^2^) are different for each simulation [63]. Such values depend of the choice of combination of 70% input data and can be varied from one to another simulation. In order to check the robustness of EDT Bagged and EDT Boosted models, thus, 1000 different arrangements of data were generated using uniform distribution and the error measurements in each case were collected. RMSE, MAE, or R^2^ errors for 1000 different simulations are plotted under scatter points in Figure 4 for training and testing parts. The variation of error measurements in the training part is rather smaller than that of the testing part in all cases, which clearly demonstrate the dependence of the predicted results on input combination. In the case of EDT Boosted model (Figure 4a–c), the training and testing part vary around the same values, i.e., RMSE = 141.21, MAE = 79.65, and R^2^ = 0.855. Regarding EDT Bagged algorithm (Figure 4d–f), the training results are RMSE = 25.82, MAE = 9.83 and R^2^ = 0.988, whereas the testing one are RMSE = 23.78, MAE = 9.14, and R^2^ = 0.990. This indicates that EDT Bagged outperforms EDT Boosted. However, both EDT Boosted and EDT Bagged algorithms possess ability to well-predict bubble dissolution time in the SLS process.

**Figure 5:**
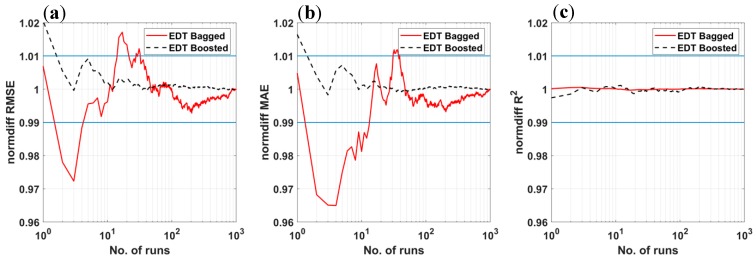
Statistical convergence analysis of (**a**) RMSE, (**b**) MAE, and (**c**) R^2^ over 1000 Monte Carlo simulations for EDT Bagged and EDT Boosted algorithms. Blue lines representing ±1% deviation around the average value.

The normalized statistical convergence analysis of the obtained results is calculated using Equation (3) and plotted in Figure 5. It is observed that over two Monte Carlo runs for EDT Boosted model are required to achieve the convergence within the 1% range around the converged values. On the contrary, RMSE and MAE criteria require at least 30 simulations to achieve converged values. For R^2^ criterion, both algorithms are converged in a 1% error range without any simulations. These results demonstrate that the two proposed AI algorithms are promising methods for the prediction of bubble dissolution time with an interesting convergence rate. Out of these, the EDT Bagged is better in term of precision than the EDT Boosted but it requires more simulations to achieve converged statistical values.

### 3.2. Sensitivity Analysis of Input Parameters

Prediction of bubble dissolution time is a complex problem in which physical and mechanical equations are highly coupled and nonlinear. Thus, the sensitivity analysis is then carried out in order to evaluate the impact of each single input parameter to the predicted output. This could be an attempt to reduce the input space if one input variable is found not affect the final prediction results. All the input parameters were successively excluded from the input space by setting the column to zero values. Monte Carlo simulations were then performed in order to quantify the influence of each parameter, thanks to the prediction performance, i.e., RMSE, MAE, and R^2^. In excluding successively I_1_, …, I_7_, a total number of 7 groups of analysis were generated and simulated with a number of 1000 runs for each group. The statistical performances (RMSE, MAE, and R^2^) of two AI models are highlighted in Table 2. Based on mean values of RMSE, MAE and R^2^ over 14,000 simulations, the EDT Bagged outperforms the EDT Boosted. Moreover, standard deviation values of EDT Bagged model are smaller than that of the EDT Boosted, indicating that the EDT Bagged algorithm is a more stable prediction technique.

Let us consider the case where input parameters I_1_ to I_7_ are successively excluded from the input space. It is observed that when I_3_ is excluded from the prediction process, highest mean values of RMSE and MAE are obtained, i.e., RMSE = 220.44, MAE = 165.46 for EDT Bagged algorithm and RMSE = 228.18, MAE = 149.14 for EDT Boosted model. Regarding R^2^, lowest mean values are obtained: R^2^ = 0.122 for EDT Bagged algorithm and R^2^ = 0.168 for EDT Boosted model. This means that without the diffusion coefficient (I_3_) in the input space, it is difficult to achieve acceptable performance of the AI prediction tools. Therefore, the diffusion coefficient (I_3_) is the most important input variable in the prediction process of bubble dissolution time. Although there is no known analytical solution to predict the bubble dissolution process in Selective Laser Sintering until now, several observations in good agreement with the results can be found in the literature. Kontopoulou et al. [8], Gogos [18] reported the kinetic of bubble shrinkage and concluded that such mechanism is mostly controlled by the diffusion of air from the bubble to the polymer melt. The distribution of the resulted dissolution time was highly distributed in the range between 20 s and 200 s. This in good agreement with this finding, as the mean value of I_3_ was rather high, i.e., 4.1 × 10^−9^ m^2^·s^−1^.

Based on the RMSE, MAE, and R^2^ values, the second and the third important input factors in the prediction process are the initial concentration (saturation rate-I_6_) and the initial bubble size (I_1_). Indeed, without I_6_, the as-obtained errors are: RMSE = 124.63, MAE = 71.64, R^2^ = 0.719 for EDT Bagged algorithm, whereas RMSE = 184.26, MAE = 101.63, R^2^ = 0.554 in case of EDT Boosted model. Besides, in excluding I_1_ out of the input space, the corresponding errors are: RMSE = 100.80, MAE = 48.86, R^2^ = 0.816 for EDT Bagged algorithm, whereas RMSE = 157.11, MAE = 87.85, R^2^ = 0.742 for EDT Boosted model. The higher values of RMSE, MAE and the lower values of R^2^ compared to that of the simulation using 7 inputs indicates the necessity of these variables in the prediction of bubble dissolution time which is in good agreement with the work of Kontopoulou et al. [8] where the authors mentioned that the air concentration and bubble initial size were found to be of great important in the bubble shrinkage process. Again, the air initial concentration distribution was taken to achieve mean value of I_6_ = 0.54, SD = 0.37, and the initial bubble size mean value I_1_ = 112.4 µm, SD = 19.37 µm. The skewness in the input space affected the bubble dissolution time to be in a range between 20 s–200 s, which was in good agreement with observations in the literature.

In our previous work [17], the contribution lies on the simulation tool for the prediction of bubble dissolution process. The polymer domain is generally considered an infinite domain in the literature, whereas we have introduced the parameter that accounting for the size of the polymer domain (I_2_). In the sensitivity analysis of this study, such parameter is found to be more important than the others (I_4_, I_5_ and I_7_). The error when excluding I_2_ are RMSE = 58.84, MAE = 26.57, R^2^ = 0.938 for EDT Bagged algorithm and RMSE = 142.84, MAE = 80.54, R^2^ = 0.843 for EDT Boosted model. The surface tension (I_4_), applied pressure (I_7_), and viscosity (I_5_) are found to be the most insensitive input parameters for the prediction problem (Table 2). The role of these parameters has also been discussed in several works relating the SLS process, such as the surface tension was neglected to derive an analytical solution [64], or its effect is negligible under certain condition, i.e., small degree of saturation [18]. The bubble shrinkage process is not influenced considerably by melt viscosity, as reported in the work of Kontopoulou et al. [8]. Last but not least, the effect of pressure is important only in case when it is applied after the formation of the bubble [8,18]. In our developed numerical tool, the pressure is considered constant during the whole SLS process [17]. Therefore, no conclusion on the effect of pressure can be deduced at this moment.

**Figure 6:**
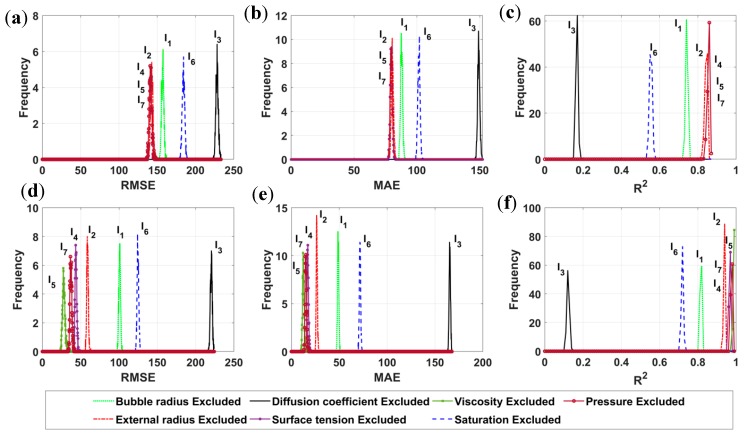
Histogram of RMSE, MAE, and R^2^ for 14,000 simulations in case of: (**a**–**c**) EDT Boosted model, and (**d**–**f**) EDT Bagged algorithm.

The histogram of RMSE, MAE, and R^2^ values of 14,000 simulations are plotted in Figure 6. All the peaks are rather narrow, demonstrating small values of standard deviation. The three most influenced parameters are easily observed, as they are separated to the rest. However, depending on the AI models, other input parameters can be detected or not. In case of EDT Boosted algorithm, the curves relating I_2_, I_4_, I_5_, and I_7_ are superimposed for RMSE, MAE and R^2^ criteria. Regarding the EDT Bagged algorithm, these input parameters can be slightly identified by RMSE and R^2^ criteria, whereas it is difficult for MAE criterion. As a conclusion, sensitivity analysis requires not only adapted error criteria but also a good prediction model that can capture the difference influence between variables.

**Figure 7:**
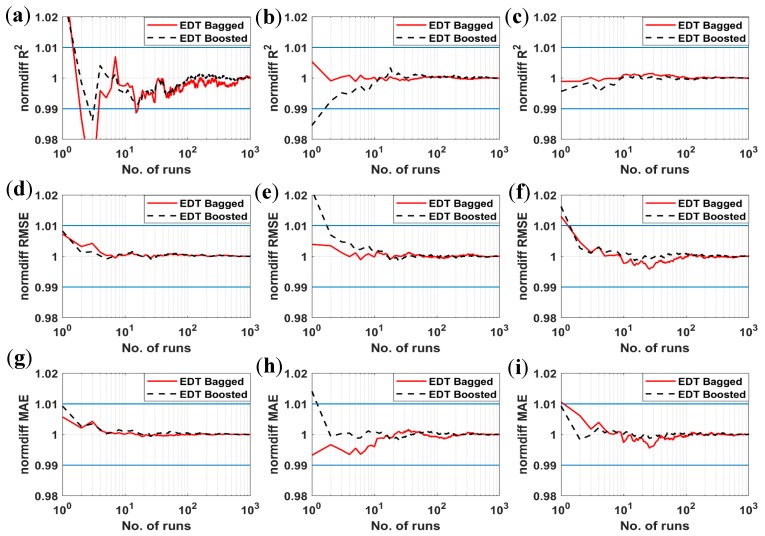
Statistical convergence analysis of RMSE, MAE, and R^2^ in case of: (**a**–**c**) diffusion coefficient excluded, (**d**–**f**) initial saturation excluded, and (**g**–**i**) initial bubble size excluded. Blue lines representing ±1% deviation around the average value.

The normalized statistical convergence analysis of the results are plotted in Figure 7 (see also Equation (3)), for the cases of three most influenced input parameters. Again, it is observed that over two Monte Carlo simulations for both EDT Boosted and EDT Bagged models are required to achieve the convergence within the 1% range around the converged values. On the contrary, for R^2^ criterion, both algorithms are converged in a 1% error range within 10 Monte Carlo simulations. These results demonstrate that the two proposed AI algorithms are potential candidates for the prediction of bubble dissolution time with an interesting convergence rate. Out of these, the EDT Bagged is better in term of precision than the EDT Boosted but it requires more simulations to achieve converged statistical values.

## 4. Conclusions

In this study, two ensemble AI models namely EDT Bagged and EDT Boosted were proposed and compared for predicting bubble dissolution time of the SLS process. A metadata of 68,644 simulated ones were created with the help of our previously developed numerical tool. They were used for generating datasets, which included input parameters (initial bubble size, external domain size, diffusion coefficient, surface tension, viscosity, initial concentration, the SLS chamber pressure) and output parameter (bubble dissolution time). Validation of the models was achieved using several criteria such as MAE, RMSE, and R^2^. Monte Carlo approach was finally used to carry out the sensitivity analysis for determination of the most important input parameter for the prediction of bubble dissolution time.

The results showed that both AI models performed well for prediction of the bubble dissolution time of the SLS process but EDT Bagged (MAE = 9.14, RMSE = 23.78, and R^2^ = 0.990) outperforms EDT Boosted (MAE = 79.62, RMSE = 141.16, and R^2^ = 0.856). The sensitivity analysis results showed that three most important input of the prediction problem were diffusion coefficient, initial concentration and bubble initial size. The present study simplified the mechanical problem of the SLS process in considering the dissolution of a single spherical gas. Therefore, a perspective of the study is to consider 2D and 3D problem to mimic the real bubble shrinkage problem. Other hybrid AI models should also be studied to improve the predictive capability. In the case of simplified approach, the computation time was estimated about 2 min for each dataset. However, the AI approaches would be more significant and useful when considering the prediction of 2D and 3D problem, where computation time would be extensively high for the generation of one dataset. Investigation of other sensitivity analysis method such as Global Sensitivity Analysis (GSA) [65] will also be a perspective of this study.

## Figures and Tables

**Table 1:** Input and output quantities used in the artificial intelligence (AI) models.

Input Parameter	Minimum	Maximum	Average	Standard Deviation
I_1_ (bubble radius, µm)	30	150	112.4	19.37
I_2_ (external radius, µm)	33	150,000	333.1	422.15
I_3_ (diffusion coefficient, m^2^·s^−1^)	0.1 × 10^−9^	50 × 10^−9^	4.1 × 10^−9^	6.79 × 10^−9^
I_4_ (surface tension, N·m^−1^)	0.0100	0.0500	0.0288	0.0144
I_5_ (viscosity, Pa·s)	100	10,000	3670.5	3701.3
I_6_ (saturation)	0.1	1	0.54	0.37
I_7_ (chamber pressure, atm)	0.25	1.5	0.88	0.41
O (bubble dissolution time, s)	20	1200	194.2	234.9

**Table 2:** Sensitivity analysis of input parameters with respect to RMSE, MAE, and R^2^ obtained after 1000 Monte Carlo simulations for each case: full simulation with 7 inputs (No excl.) and excluding successively I_1_ to I_7_ (I_1,_ …, I_7_ excl.).

Criteria	Input Excl.	EDT Bagged				EDT Boosted			
		Min	Max	Mean	SD	Min	Max	Mean	SD
RMSE	No excl.	19.20	28.27	23.78	1.26	135.86	146.70	141.16	1.79
	I_1_ excl.	97.48	104.97	100.80	1.15	153.04	161.99	157.11	1.49
	I_2_ excl.	56.13	63.04	58.84	1.02	138.32	150.13	142.84	1.68
	I_3_ excl.	215.67	224.28	220.44	1.18	222.84	223.37	228.18	1.61
	I_4_ excl.	40.06	48.21	43.71	1.12	136.29	147.91	141.28	1.69
	I_5_ excl.	23.50	36.27	27.83	1.70	136.29	147.91	141.28	1.69
	I_6_ excl.	120.65	128.45	124.63	1.12	178.98	189.90	184.26	1.67
	I_7_ excl.	33.37	41.85	37.24	1.28	136.29	147.91	141.28	1.69
MAE	No excl.	7.93	10.82	9.14	0.47	77.30	82.33	79.62	0.91
	I_1_ excl.	46.87	51.16	48.86	0.69	85.76	90.81	87.85	0.82
	I_2_ excl.	24.95	31.25	26.57	0.63	78.39	83.60	80.54	0.85
	I_3_ excl.	163.07	167.72	165.46	0.73	145.98	152.37	149.14	0.88
	I_4_ excl.	15.13	20.76	17.15	0.75	77.41	82.77	79.65	0.86
	I_5_ excl.	10.15	15.99	12.54	0.92	77.41	82.77	79.65	0.86
	I_6_ excl.	69.15	73.83	71.64	0.71	98.73	104.90	101.63	0.90
	I_7_ excl.	13.24	19.13	15.18	0.83	77.41	82.77	79.65	0.86
R^2^	No excl.	0.986	0.993	0.990	0.001	0.832	0.870	0.856	0.006
	I_1_ excl.	0.802	0.827	0.816	0.004	0.721	0.758	0.742	0.005
	I_2_ excl.	0.929	0.943	0.938	0.002	0.808	0.858	0.843	0.007
	I_3_ excl.	0.100	0.144	0.122	0.006	0.153	0.183	0.168	0.005
	I_4_ excl.	0.960	0.971	0.966	0.002	0.831	0.872	0.855	0.006
	I_5_ excl.	0.978	0.990	0.987	0.002	0.831	0.872	0.855	0.006
	I_6_ excl.	0.704	0.731	0.719	0.004	0.533	0.573	0.554	0.007
	I_7_ excl.	0.970	0.980	0.975	0.002	0.831	0.872	0.855	0.006
